# Intestinal absorption of glucose in mice as determined by positron emission tomography

**DOI:** 10.1113/JP275934

**Published:** 2018-06-05

**Authors:** Monica Sala‐Rabanal, Chiara Ghezzi, Bruce A. Hirayama, Vladimir Kepe, Jie Liu, Jorge R. Barrio, Ernest M. Wright

**Affiliations:** ^1^ Department of Physiology David Geffen School of Medicine at UCLA University of California Los Angeles CA 90095‐1571 USA; ^2^ Department of Cell Biology and Physiology and Center for the Investigation of Membrane Excitability Diseases (CIMED) Washington University St Louis MO 63110 USA; ^3^ Department of Molecular and Medical Pharmacology David Geffen School of Medicine at UCLA University of California Los Angeles CA 90095‐1735 USA

**Keywords:** intestine, absorption, PET

## Abstract

**Key Points:**

- The goal was to determine the importance of the sodium–glucose cotransporter SGLT1 and the glucose uniporter GLUT2 in intestinal glucose absorption during oral glucose tolerance tests (OGTTs) in mice.
- Glucose absorption was determined in mice using positron emission tomography and three non‐metabolizable glucose probes: one specific for SGLTs, one specific for GLUTs, and one a substrate for both SGLTs and GLUTs.
- Absorption was determined in wild‐type, *Sglt1^−/−^* and *Glut2^−/−^* mice.
- Gastric emptying was a rate‐limiting step in absorption.
- SGLT1, but not GLUT2, was important in fast glucose absorption.
- In the absence of SGLT1 or GLUT2, the oral glucose load delivered to the small intestine was slowly absorbed.
- Oral phlorizin only inhibited the fast component of glucose absorption, but it contributed to decreasing blood glucose levels by inhibiting renal reabsorption.

**Abstract:**

The current model of intestinal absorption is that SGLT1 is responsible for transport of glucose from the lumen into enterocytes across the brush border membrane, and GLUT2 for the downhill transport from the epithelium into blood across the basolateral membrane. Nevertheless, questions remain about the importance of these transporters *in vivo*. To address these questions, we have developed a non‐invasive imaging method, positron emission tomography (PET), to monitor intestinal absorption of three non‐metabolized glucose tracers during standard oral glucose tolerance tests (OGTTs) in mice. One tracer is specific for SGLTs (α‐methyl‐4‐[^18^F]fluoro‐4‐deoxy‐d‐glucopyranoside; Me‐4FDG), one is specific for GLUTs (2‐deoxy‐2‐[^18^F]fluoro‐d‐glucose; 2‐FDG), and one is a substrate for both SGLTs and GLUTs (4‐deoxy‐4‐[^18^F]fluoro‐d‐glucose; 4‐FDG). OGTTs were conducted on adult wild‐type, *Sglt1^−/−^* and *Glut2^−/−^* mice. In conscious mice, OGTTs resulted in the predictable increase in blood glucose that was blocked by phlorizin in both wild‐type and *Glut2^−/−^* animals. The blood activity of both Me‐4FDG and 4‐FDG, but not 2‐FDG, accompanied the changes in glucose concentration. PET imaging during OGTTs further shows that: (i) intestinal absorption of the glucose load depends on gastric emptying; (ii) SGLT1 is important for the fast absorption; (iii) GLUT2 is not important in absorption; and (iv) oral phlorizin reduces absorption by SGLT1, but is absorbed and blocks glucose reabsorption in the kidney. We conclude that in standard OGTTs in mice, SGLT1 is essential in fast absorption, GLUT2 does not play a significant role, and in the absence of SGLT1 the total load of glucose is slowly absorbed.

## Key Points

- The goal was to determine the importance of the sodium–glucose cotransporter SGLT1 and the glucose uniporter GLUT2 in intestinal glucose absorption during oral glucose tolerance tests (OGTTs) in mice.
- Glucose absorption was determined in mice using positron emission tomography and three non‐metabolizable glucose probes: one specific for SGLTs, one specific for GLUTs, and one a substrate for both SGLTs and GLUTs.
- Absorption was determined in wild‐type, *Sglt1^−/−^* and *Glut2^−/−^* mice.
- Gastric emptying was a rate‐limiting step in absorption.
- SGLT1, but not GLUT2, was important in fast glucose absorption.
- In the absence of SGLT1 or GLUT2, the oral glucose load delivered to the small intestine was slowly absorbed.
- Oral phlorizin only inhibited the fast component of glucose absorption, but it contributed to decreasing blood glucose levels by inhibiting renal reabsorption.

## Introduction

Glucose, in the form of complex carbohydrates, constitutes a major source of calories in the human diet, about 250 g per day in a 70 kg adult. All of the ingested glucose is normally absorbed in the intestine, and the molecular mechanisms are thought to be well understood. Active absorption occurs across the mature enterocytes lining the small intestine, first, by the sodium glucose cotransporter (SGLT1) in the brush border membrane, and second, by a glucose uniporter in the basolateral membrane (GLUT2) (Wright *et al*. 2011). Nevertheless, questions still remain about the relative importance of these transport proteins in glucose absorption *in vivo* after a meal in both humans and rodents, especially at high glucose loads.

The oral glucose tolerance test (OGTT) is an important tool in evaluating glucose absorption, although it is primarily used to assess insulin regulation of blood glucose. In such tests, a standard ‘meal’, i.e. 1–2 g of glucose per kg body weight, is administered as a bolus of 1–2 m glucose and the changes in plasma glucose are monitored as a function of time. However, it is a non‐trivial task to interpret the data with respect to intestinal absorption owing to the complex and often interacting processes controlling plasma glucose levels. These include gastric emptying, glucose absorption, uptake, metabolism and release in multiple organs, as well as regulation of these processes by neural and endocrine inputs. In animals, it has been popular to use more invasive *in vivo* techniques, such as perfusion of isolated intestinal loops (Debnam & Levin, 1975), where absorption, measured by glucose, galactose or α‐methyl‐d‐glucopyranoside (αMDG) disappearance from the perfusate, occurred via two components, namely a saturable process at low concentrations and a non‐saturable process at high concentrations. Studies by Kellett (Kellett, 2001; Kellett & Brot‐Laroche, 2005) led to the controversial hypothesis that the ‘diffusion component’ in mice is carried out by GLUT2 recruited into the brush border membrane from the cytoplasm. It is noteworthy that αMDG is not a substrate for GLUT2.

In this work, we have turned to a novel method, positron‐emission tomography (PET), to follow the dynamics of glucose absorption during OGTTs in mice using non‐metabolizable SGLT‐ and GLUT‐specific probes (Sala‐Rabanal *et al*. 2016). A major advantage of PET is that it is a non‐invasive technique to record the distribution of the glucose tracers throughout the whole mouse with high spatial (2 mm) and temporal resolution. In addition to wild‐type mice, we have also used *Sglt1^−/−^* and *Glut2^−/−^* mice (Sala‐Rabanal *et al*. 2016).

Our results show that the rate of gastric emptying limits glucose intestinal absorption, SGLT1 has a key role in the fast absorption of glucose from the duodenum, but neither SGLT1 nor GLUT2 is essential for the total absorption of glucose over the duration of OGTTs.

## Methods

### Animals

All animal procedures followed guidelines approved by the University of California Chancellor's Committee on Animal Research and the National Institutes of Health. We understand the ethical principles under which *The Journal of Physiology* operates and our work complies with the animal ethics policy and checklist as outlined recently (Grundy, 2015). Generally, experiments were carried out on mice used in our prior study to determine the i.v. biodistribution of α‐methyl‐4‐[^18^F]fluoro‐4‐deoxy‐d‐glucopyranoside (Me‐4FDG), 2‐deoxy‐2‐[^18^F]fluoro‐d‐glucose (2‐FDG) and 4‐deoxy‐4‐[^18^F]fluoro‐d‐glucose (4‐FDG) (Sala‐Rabanal *et al*. 2016). The animals include male and female wild‐type C57Bl/6 mice from The Jackson Laboratory (Bar Harbor, ME, USA); and female *Glut2^–/–^* mice and male *Sglt1^–/–^* mice on a C57Bl/6 background (Thorens *et al*. 2000; Gorboulev *et al*. 2012). The control experiments for the *Sglt1^–/–^* and *Glut2^−/−^* mice were conducted on the same‐sex wild‐type mice. Preliminary studies did not reveal differences between sexes, or between mouse strains. For some experiments, we used wild‐type C57Bl/6 mice with indwelling duodenal catheters (Taconic Biosciences, Hudson, NY, USA). All animals were housed at the UCLA Division of Laboratory Animal Medicine facilities, and maintained on a 12 h light–dark cycle, with food and water available *ad libitum*. *Sglt1^–/–^* mice were kept on a low carbohydrate diet (Teklad TD08212; Harlan, Indianapolis, IN, USA) to avoid diarrhoea caused by glucose–galactose malabsorption (Wright *et al*. 2001; Gorboulev *et al*. 2012). Prior to each study, mice were fasted overnight in cages with free access to water. Experiments were conducted between 10.00 and 14.00 h. At the termination of the studies, animals were killed by terminal exsanguination under deep isoflurane anaesthesia (Abbott Laboratories, Chicago, IL, USA), followed by thoracotomy.

### Radiochemistry, probe metabolism and selectivity

2‐FDG was obtained from the UCLA Biomedical Cyclotron, and Me‐4FDG and 4‐FDG were synthesized as described (Yu *et al*. 2010, 2013; Wright *et al*. 2014; Sala‐Rabanal *et al*. 2016) from the corresponding acetylated galactose triflate by radiofluorination with cyclotron‐produced [^18^F]fluoride, followed by deacetylation. Probes had high chemical and radiochemical purities (> 97%) and specific radioactivity (> 2000 Ci mmol^−1^) (Wright *et al*. 2014). As previously demonstrated, 2‐FDG is a preferred substrate for GLUTs (*K*_0.5_ 7_ _mm for hGLUT2 and > 300 mm for hSGLT1), Me‐4FDG is a preferred substrate for SGLTs (*K*_0.5_ 0.06 mm for hSGLT1 and 14 mm for hGLUT2), and 4‐FDG is a substrate for both GLUTs and SGLTs (*K*_0.5_ 14_ _mm for hGLUT2 and 0.1 mm for hSGLT1). Neither Me‐4FDG nor 4‐FDG is phosphorylated by hexokinase *in vitro* or metabolized *in vivo*, whereas 2‐FDG is phosphorylated intracellularly by hexokinase to 2‐FDG‐6‐phosphate (Yu *et al*. 2010; Sala‐Rabanal *et al*. 2016).

### Simultaneous OGTT and blood tracer determination

Mice (20‐40 g) were given 1 mg g^−1^ phlorizin (100–200 μl of a 20% solution in propylene glycol) or the appropriate volume of excipient as a control, directly into the stomach, using a bulb‐tipped gastric gavage needle. Twenty minutes later, this was followed by gavage of 2 mg g^−1^ glucose (100–200 μl of a 1.3 m glucose solution in water containing 300–500 μCi Me‐4FDG, 2‐FDG or 4‐FDG). Blood glucose levels were determined immediately before phlorizin (or vehicle) pre‐treatment (i.e. fasting baseline), and at selected times after delivery of the glucose/tracer bolus in conscious mice (i.e. at 15, 30, 45, 60 and 120 min). Blood (1 μl) was obtained from a small tail vein puncture and assayed with a commercial glucose meter (Contour; Bayer, Leverkusen, Germany). Subsequently, the glucose meter strips were counted for fluorine‐18 activity in a gamma counter (1480 Wizard; PerkinElmer, Waltham, MA, USA). Animals were lightly anaesthetized with 1% isoflurane prior to the gavage interventions, but otherwise allowed to roam freely in their holding cage for the duration of the experiment, and handled for less than 1 min at each blood collection.

### *In vivo* microPET scanning and data analysis

Experiments were performed at the Preclinical Imaging Technology Centre of the UCLA Crump Institute for Molecular Imaging. A MicroPET Focus 220 scanner (CTI Concorde Microsystems, Knoxville, TN, USA) was used for PET data acquisition where all data were corrected automatically for ^18^F decay. To determine the absorption and tissue distribution of orally delivered glucose radiotracers in mice, animals were lightly anaesthetized with 1% isoflurane and administered ∼300 μCi 2‐FDG, Me‐4FDG or 4‐FDG by orogastric gavage. Where indicated, the tracers were delivered in a solution containing 2 mg g^−1^ glucose, following pre‐treatment with 1 mg g^−1^ phlorizin in propylene glycol or vehicle as described above. For conscious studies (Figs 4 and 6, and Table 1), mice were returned to their holding cage after gavage and allowed to roam freely. Ten minutes before the scan was scheduled to start, mice were anaesthetized in a heated induction box by inhalation of 2% isoflurane in 100% oxygen, positioned on a heated custom PET‐CT small animal holder, which allows for continuous anaesthesia (Suckow *et al*. 2009), and PET‐scanned for 10 min. For dynamic microPET studies (Figs 5, 7 and 8), anaesthetized animals were placed on the PET‐CT holder immediately after oral gavage and a 1 h PET scan promptly started; a delay of approximately 2 min between tracer delivery and scan initiation has been taken into account when representing the data. To assess absorption and distribution of the tracers when delivered directly to the intestine (Fig. 9), mice with a duodenal catheter were anaesthetized, and positioned on the scanning bed. A 1 h PET scan was initiated after infusion into the duodenum of 200 μl of a 0.9% NaCl solution containing 200 μCi Me‐4FDG and 5 mm α‐MDG; or 200 μCi 2‐FDG and 5 mm 2‐deoxy‐d‐glucose (2‐DG); or 200 μCi 4‐FDG and 5 mm glucose.

**Table 1:** Bio‐distribution of orally delivered glucose PET tracers Me‐4FDG, 2‐FDG and 4‐FDG in mice

Percentage of absorbed tracer in target organ						
	Me‐4FDG		2‐FDG		4‐FDG	
Organ	Wild‐type	*Glut2^−/−^*	Wild‐type	*Glut2^−/^* ^−^	Wild‐type	*Glut2^−/−^*
Brain	0.29 ± 0.04^b^	0.1, 0.3^b^	3.7 ± 0.7^ab^	4.9 ± 1.2^a^	4.0 ± 1.5^a,b^	2.5 ± 0.5^a,b^
Heart	1.4 ± 0.2	1.1, 1.3	3.1 ± 0.7	2.1 ± 0.5	2.0 ± 0.6	1.7 ± 0.3
Muscle	0.30 ± 0.03^a^	0.3, 0.2^a,b^	0.11 ± 0.03^b^	0.13 ± 0.02^b^	0.13 ± 0.03^b^	0.15 ± 0.03^a,b^
Kidney	0.9 ± 0.1^b^	6, 5^a^	1.1 ± 0.2^b^	3.8 ± 0.9^a^	1.4 ± 0.3^b^	5.7 ± 0.9^a^
Bladder	1.4 ± 0.4^b^	19, 17^a,b^	19 ± 3^a^	14 ± 3^a,b^	7 ± 2^a,b^	13 ± 3^a,b^

Data are from the same experiments as in Fig. 4. Results are mean ± SEM of 6–14 experiments except for Me‐4FDG in *Glut2^−/−^* mice, where the individual values are shown instead. For each target organ (brain, heart, muscle, kidney, or urinary bladder), significant differences in tracer uptake between wild‐type and Glut2‐null animals are indicated by different lowercase letters (*P* < 0.05, two way ANOVA and Tukey's test). *Kidney* refers to the right kidney, and *Muscle* refers to a 64 mm^3^ spherical VOI from the inner right thigh (the same in all mice).

For dynamic scans of the anaesthetized whole mouse, the acquired data were binned into 25 image frames (3 × 20, 4 × 30, 5 × 60, 5 × 120, 3 × 180, 3 × 300, and 2 × 600 s for Figs 5, 8 and 9; or 1 × 1.75, 11 × 0.5, 1 × 1, 1 × 8.75, 1 × 24, 1 × 106, 1 × 240, 1 × 380, 1 × 240, 1 × 380, 1 × 500, 1 × 570, 1 × 675, 1 × 725 and 1 × 355 s for Fig. 7).

After completion of the PET data acquisition, 10 min computed tomography (CT) scans were performed to provide anatomical information, using a MicroCAT II X‐ray Tomograph (ImTek Inc., Knoxville, TN, USA). PET images were reconstructed using Fourier rebinning and a filtered back‐projection algorithm (Kreissl *et al*. 2011; Wong *et al*. 2013), and microCT images were co‐registered with microPET data for attenuation correction (Chow *et al*. 2005). AMIDE software (Loening & Gambhir, 2003) was used for image display and volume‐of‐interest (VOI) analysis.

**Figure 1:**
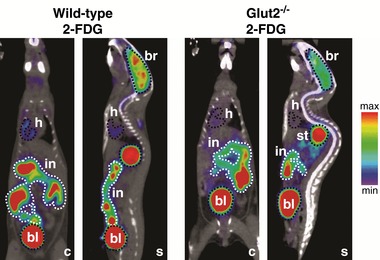
Distribution of 2‐FDG in representative wild‐type and *Glut2^–/–^* mice 60 min after gavage to illustrate the determination of gastric emptying and intestinal absorption For each mouse, coronal (c) and sagittal (s) sections (0.2 mm thick) are shown, and the areas corresponding to the three‐dimensional volumes of interest (VOIs) for intestine (in), stomach (st), brain (br), heart (h) and urinary bladder (bl) are delineated. Images are displayed according to the NIH intensity scale for tracer activity, from red (highest), through green (intermediate) to purple (lowest). VOI analysis was performed using Amide software. Gastric emptying was estimated from the difference between the 2‐FDG measured in the whole‐body VOI and the amount remaining in the stomach, and intestinal absorption was estimated from the amount of 2‐FDG in the whole body minus the sum of the amounts detected in the stomach and the small intestine.

Gastric emptying and intestinal absorption were estimated by determining tracer concentration in stomach and small intestine as a function of time. The approach is explained in Fig. 1 where the distribution of 2‐FDG in wild‐type and *Glut2^−/−^* mice 60 min after gavage is shown. In each experiment, volumes of interest (VOI) for the whole mouse, stomach (st), intestine (in), brain (br) and bladder (bl) were drawn, and the activity of tracer (μCi) in each VOI at 60 min was estimated using Amide software (Loening & Gambhir, 2003). Gastric emptying was obtained from the difference between the initial and final activities in the stomach VOI; the initial amount gavaged was estimated from the activity detected in the whole‐mouse VOI, which was within a small percentage of that measured in the administered gavage solution (100–500 μCi). Intestinal absorption of the tracer that was emptied into the stomach was estimated from the total activity in the whole body minus the sum of that remaining in the stomach and in the intestine. The approach is validated by the accumulation of absorbed tracer in the brain, bladder and heart following oral delivery of 2‐FDG (Fig. 1). In these examples, 125 μCi of 2‐FDG in 200 μl of 1.3 m glucose (260 μmol of glucose) was injected into the stomach at the start of the OGTT. After 60 min, 24 (wild‐type; Fig. 1, left) and 46 μCi (*Glut2^−/−^*; Fig. 1, right) remained in the stomach, 20 and 15 μCi in the intestine; 10 and 8 μCi accumulated in brain and 30 and 27 μCi in the urinary bladder. Therefore in 60 min, 81% and 63% of the initial amount in the stomach (211 and 164 μmol of glucose) was emptied into the intestine, 80% and 92% of the amount entering the intestine was absorbed (169 and 151 μmol), 13% of the amount absorbed was taken up into the brain, and 30% and 27% (51 and 41 μmol of glucose) was excreted into the urinary bladder. Given the specific activity of the gavage solution, i.e. 260 μmol and 125 μCi, gastric emptying, intestinal absorption, renal excretion and uptake into each organ may also be expressed in moles h^−1^.

### Statistical analysis

**Figure 2:**
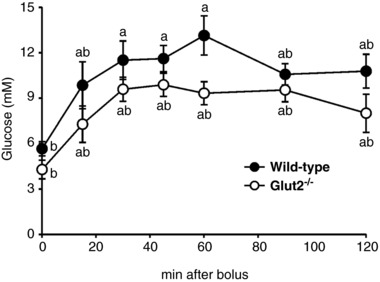
Standard OGTTs in wild‐type and in *Glut2^–/–^* mice Time course of blood glucose levels after orogastric gavage of 2 mg g^−1^ of glucose. Data are means ± SEM from 10 (wild‐type) or 4 (*Glut2^–/–^*) mice. Groups that share letters are statistically similar (‘a’ and ‘ab’, or ‘b’ and ‘ab’), whereas those not sharing any letters (‘a’ and ‘b’) are significantly different (*P* < 0.05, one‐way ANOVA and Tukey's test).

SigmaPlot 10.0 (Systat Software, San Jose, CA, USA) and CorelDRAW X8 18.1 (Corel Corp., Mountain View, CA, USA) were used for figure preparation. Unless otherwise noted, data are shown as mean ± SEM of at least three experiments. Statistical analyses were performed with Origin (OriginLab Corp., Northampton, MA, USA). The differences in blood glucose levels between wild‐type and *Glut2^−/−^* in OGTTs (Fig. 2) were tested by means of one‐way ANOVA followed by *post hoc* all‐pairwise Tukey's test. The differences in 60 min conscious gastric emptying, absorption and accumulation into target organs, i.e. brain, heart, muscle, kidney and urinary bladder (Fig. 4 and Table 1) were evaluated by means of two‐way ANOVA followed by the Tukey's test, using tracer (Me‐4FDG, 2‐FDG or 4‐FDG) and genotype (wild‐type or *Glut2^−/−^*) as independent variables. Where appropriate, the results of the statistical analysis are indicated by a lowercase letter system, whereby groups that share letters are statistically similar (for example, ‘a’ and ‘ab’, or ‘b’ and ‘ab’), whereas those not sharing any letters are significantly different (*P* < 0.05; for example, ‘a’ and ‘b’). Additionally, where indicated, Student's *t* test was applied to examine differences between treatments.

## Results

### OGTT

**Figure 3:**
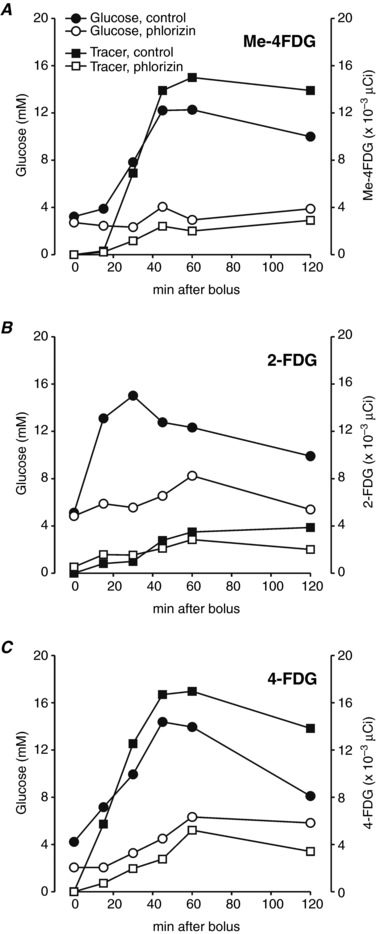
Time course of blood glucose concentration and activity of glucose PET tracers Me‐4FDG, 2‐FDG and 4‐FDG following oral delivery in mice Following pre‐treatment with SGLT1 inhibitor phlorizin (1 mg g^−1^) or with phlorizin‐free excipient (control), fasted wild‐type mice were administered an oral dose of glucose (2 mg g^−1^) with 300 μCi of Me‐4FDG (*A*), 2‐FDG (*B*) or 4‐FDG (*C*). Blood glucose concentration (circles) and radiotracer activity (squares) were simultaneously monitored over the next 2 h in control (filled symbols) and phlorizin treated (open symbols) mice. The fasting blood samples (‘0 min’) were taken immediately before phlorizin or vehicle pre‐dosing. Data are from individual experiments where phlorizin and control mice were tested side by side, and results were confirmed in at least one additional set of experiments with different mice for each tracer.

Following an overnight fast and just prior to OGTT experiments, blood glucose was consistently between 4 and 6 mm (see Figs 2 and 3), that is, within the normal range. After gavage with 200 μl of 2 mg g^−1^ of glucose (1.3 M), the blood glucose level rose to peak at 12–14 mm within 60 min, and then declined slowly towards baseline (Figs 2 and 3). When mice were pre‐treated with 1 mg g^−1^ phlorizin, the blood glucose concentration remained relatively flat at the fasting level, but it did increase slowly by up to 3 mm (Fig. 3). As phlorizin is a high affinity, specific competitive inhibitor of SGLTs, at least at low concentrations (< 100 μm), this has been taken as evidence for the importance of SGLT1 in glucose absorption. OGTTs were also conducted on *Glut2^−/−^* mice with similar results (Fig. 2) and there were no statistically significant differences between blood glucose levels at each time point, except for a small difference in the fasting levels. When the data were normalized to the fasting blood glucose levels, the data points for the two studies overlapped (not shown).

Simultaneous evaluation of Me‐4FDG, 2‐FDG and 4‐FDG levels in blood during the OGTTs show that Me‐4FDG and 4‐FDG activity in control and phlorizin‐treated mice closely followed the blood glucose levels (Fig. 3 *A* and *C*). This was not the case for 2‐FDG (Fig. 3 *B*), where the activity in blood increased slowly in both the presence and absence of phlorizin, at rates comparable to those for Me‐4FDG and 4‐FDG in the presence of phlorizin. Our interpretation is that Me‐4FDG and 4‐FDG are tracers for glucose absorption *in vivo*, but 2‐FDG is not. Furthermore, given that Me‐4FDG is not a preferred substrate for GLUT2, and 2‐FDG is not a preferred substrate for SGLT1 (Sala‐Rabanal *et al*. 2016), these results point to an important role of SGLT1, and a minor role of GLUTs in glucose absorption during OGTTs in mice.

### Gastric emptying, intestinal absorption and tissue distribution of orally delivered glucose PET tracers in conscious mice

**Figure 4:**
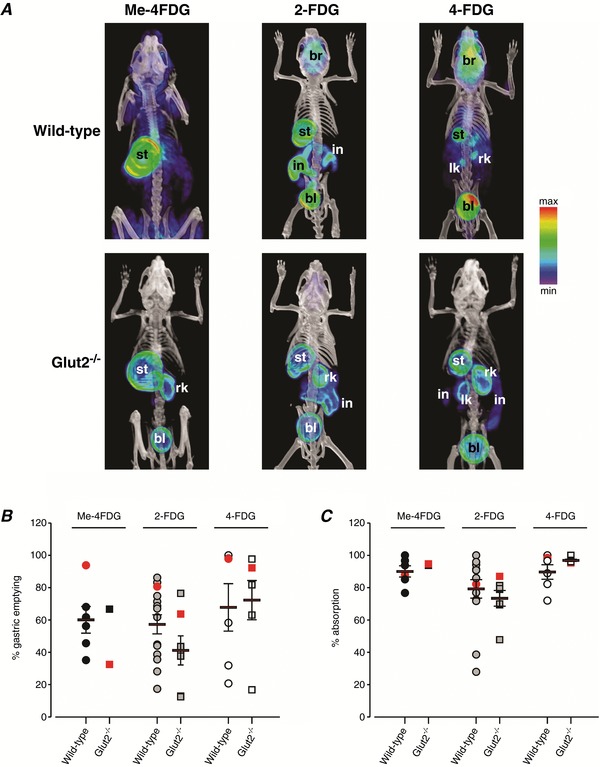
Absorption of orally delivered glucose PET tracers Me‐4FDG, 2‐FDG and 4‐FDG in mice Three hundred microcuries of Me‐4FDG, 2‐FDG or 4‐FDG was administered by oral gavage into the stomach of conscious control or *Glut2^–/–^* mice, and 10 min PET scans were performed after 60 min. *A*, volumetric renderings of representative co‐registered microPET and CT scans; PET images in this and subsequent figures are displayed according to the NIH intensity scale for tracer activity, from red (highest), through green (intermediate) to purple (lowest). Stomach (st), intestine (in), brain (br), urinary bladder (bl), left kidney (lk) and right kidney (rk) are indicated where visible. *B* and *C*, gastric emptying (*B*) and intestinal tracer absorption (*C*) 60 min after oral bolus administration in experiments as in *A*. Symbols represent data from individual experiments; red symbols are for the mice shown in *A*. Except for Me‐4FDG in *Glut2^–/–^* mice, where only two experiments were performed, bars indicate the mean ± SEM of 6–14 experiments. No significant differences in gastric emptying (*P* = 0.4319) or in tracer absorption (*P* = 0.766) were found between tracers or genotypes (two‐way ANOVA).

Preliminary studies indicated that gastric emptying was similar in conscious and isoflurane‐anaesthetized mice. In conscious mice, the biodistribution of Me‐4FDG, 2‐FDG and 4‐FDG 60 min after oral gavage was determined from 10 min microPET scans in both wild‐type and *Glut2^−/−^* mice (Fig. 4 and Table 1). The percentage of each tracer remaining in the stomach and the percentage of tracer absorbed from the intestine into the body are summarized in Fig. 4 *B* and 4 *C*. On average, gastric emptying by 1 h was ∼60% for all tracers, but this was highly variable from one mouse to another, i.e. 15–100% (Fig. 4 *B*). In both control and *Glut2^−/−^* mice, absorption of the Me‐4FDG and 4‐FDG that were delivered into the small intestine was virtually complete (∼90%) 60 min after oral delivery, whereas absorption of 2‐FDG was slightly lower, i.e. ∼80% on average, and more variable in both sets of mice. In the small intestine, 2‐FDG activity was apparent in both wild‐type and *Glut2^−/−^* mice while Me‐4FDG and 4‐FDG were visible only in the *Glut2^−/−^* mice (Fig. 4 *A*).

As summarized in Table 1, the biodistribution of absorbed Me‐4FDG, 2‐FDG and 4‐FDG in the mice shown in Fig. 4 was similar to that reported after i.v. injection of the tracers into the body (Sala‐Rabanal *et al*. 2016). Thus, 2‐FDG and 4‐FDG were taken up into the brain, heart, skeletal muscle and urinary bladder of wild‐type and *Glut2* ^−/−^ animals to a similar extent, while again very little Me‐4FDG activity was detected in the brain for either group. Consistent with our results in i.v. injected mice (Sala‐Rabanal *et al*. 2016), there were no significant differences in 2‐FDG uptake into liver between wild‐type and *Glut2^−/−^* mice, i.e. as percentage of absorbed tracer, 0.26 ± 0.05 (wild‐type, *n* = 13) and 0.42 ± 0.11 (*Glut2^−/−^*, *n* = 6), as measured in a 64 mm^3^ spherical VOI in the right lobe (*P* = 0.11, Student's *t* test). Similar results have been reported for liver in another 2‐FDG *Glut2^–/–^* mouse model (Schmitt *et al*. 2017). In wild‐type mice, only ∼1% of the absorbed Me‐4FDG was detected in the urinary bladder, and also ∼1% of the absorbed 2‐FDG, 4‐FDG or Me‐4FDG was found in kidney. In *Glut2^−/−^* mice, accumulation of 2‐FDG, 4FDG and Me‐4FDG in kidney, and notably, excretion of Me‐4FDG were increased (Table 1).

### Effect of phlorizin in Me‐4FDG and 4‐FDG absorption and excretion

**Figure 5:**
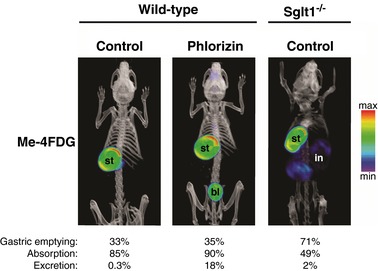
Me‐4FDG absorption is reduced in *Sglt1^−^^/^^−^* mice, but not in phlorizin‐treated wild‐type mice Glucose (2** **mg g^−1^) in presence of 300 μCi Me‐4FDG was administered by oral gavage into the stomach of anaesthetized wild‐type or *Sglt1^–/–^* mice, and continuous PET data were acquired over the following 62 min; a CT scan was performed at the end of the dynamic PET scan. Wild‐type mice were pre‐treated with 1 mg g^−1^ phlorizin, or with phlorizin‐free vehicle, as described above. Volumetric renderings of end‐point co‐registered images for representative mice are shown, and their values for gastric emptying, Me‐4FDG absorption and excretion are given at the bottom. PET images are displayed as in Fig. 4 *A;* stomach (st), intestine (in) and urinary bladder (bl) are indicated where visible. PET images are displayed according to the NIH intensity scale for tracer activity, from red (highest), through green (intermediate) to purple (lowest).

Figure 5 shows 1 h Me‐4FDG PET images of wild‐type OGTT mice treated with excipient (control, left panel) or with phlorizin (middle), in comparison with an *Sglt1^–/–^* OGTT mouse assayed under control conditions. In both wild‐type mice, about two‐thirds of the oral load remained in the stomach after 1 h, but 85–90% of the sugar delivered into the small intestine was absorbed. The most notable difference between these two treatments was the significant excretion of Me‐4FDG into the urinary bladder of the phlorizin‐treated animal; this suggests that phlorizin was absorbed intact, filtered at the glomeruli, and then inhibited Me‐4FDG reabsorption by SGLTs in the proximal tubule. In the *Sglt1^−/−^* mouse, 71% of the initial amount of tracer in the stomach was emptied into the intestine in 1 h, and 49% of that was absorbed; however, unlike in the case of the phlorizin‐pre‐treated wild‐type mouse, only 2% of the absorbed sugar was excreted into the urinary bladder. This is consistent with the fact that SGLT2, and not SGLT1, is largely responsible for glucose reabsorption in the kidney.

**Figure 6:**
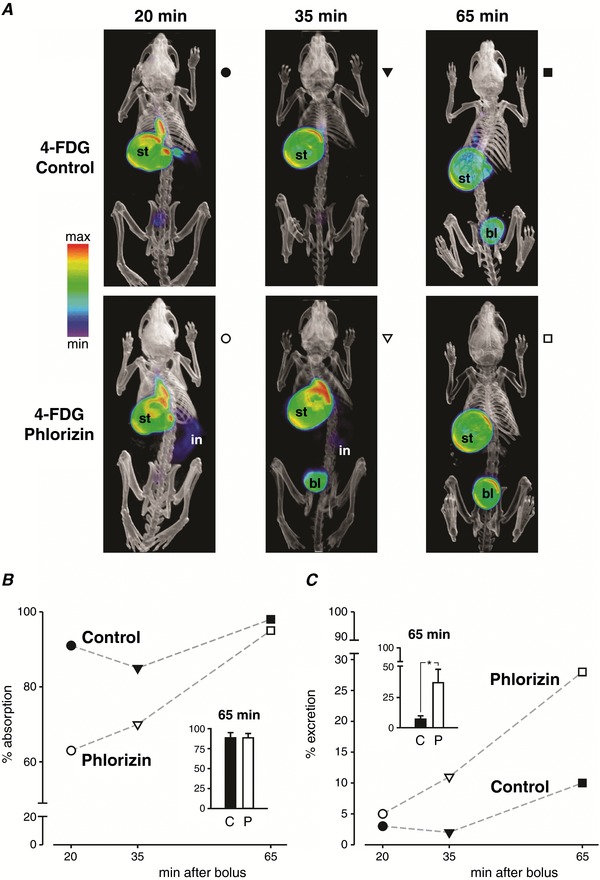
Phlorizin delays absorption and increases excretion of 4‐FDG in mice Following pre‐treatment with phlorizin, or with phlorizin‐free vehicle (control), 2 mg g^−1^ glucose in presence of 300 μCi 4‐FDG was administered by oral gavage into the stomach of conscious wild‐type mice, and brief 10 min PET scans were performed 10, 25 or 55 min later. *A*, volumetric renderings of co‐registered images in three control (top) and three phlorizin‐treated (bottom) mice treated side‐by‐side and scanned sequentially as appropriate; results were confirmed in at least one additional trial including all time points. Stomach (st), intestine (in) and urinary bladder (bl) are indicated where visible. *B* and *C*, 4‐FDG absorption (*B*) and excretion (*C*) for the mice shown in *A*. Results from each mouse are represented by a unique symbol, which is also shown to the side of each panel in *A*. Data points are joined by dashed lines for illustrative purposes only. Insets, mean ± SEM of 4‐FDG absorption (*B*) and excretion (*C*) at 65 min, in three control (C) and three phlorizin‐treated (P) mice. *P < 0.05, Student's *t* test. PET images are displayed according to the NIH intensity scale for tracer activity, from red (highest), through green (intermediate) to purple (lowest).

A time course of absorption and excretion of orally delivered 4‐FDG in conscious wild‐type OGTT mice pre‐dosed with vehicle (control) or with phlorizin is shown in Fig. 6. In both groups, 65 min after bolus administration virtually all the 4‐FDG that entered the intestine had been absorbed, but in phlorizin‐treated mice absorption was initially delayed. Thus, in control mice, over 90% of the 4‐FDG was already absorbed at 20 min, whereas in phlorizin‐treated mice absorption was ∼60% at 20 min and ∼70% at 35 min (Fig. 6 *B*). Conversely, 4‐FDG excretion into the urinary bladder was progressively increased in phlorizin‐treated mice, up to ∼35% at 65 min in contrast to ∼7% in control mice (Fig. 6 *C*). Together with the results shown in Fig. 5, this suggests that phlorizin is absorbed intact from the intestine and inhibits Me‐4FDG and 4‐FDG reabsorption in the kidney.

### Time dependence of glucose PET tracer intestinal absorption

**Figure 7:**
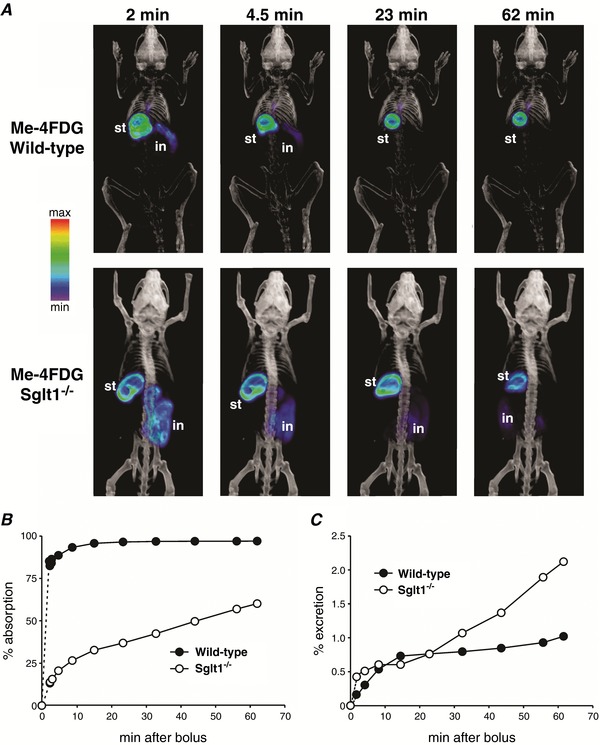
Dynamic Me‐4FDG microPET scans of control and *Sglt1^−^^/^^−^* mice undergoing OGTTs Glucose (2** **mg g^−1^) in presence of 300 μCi Me‐4FDG was administered by oral gavage into the stomach of anaesthetized wild‐type or *Sglt1^–/–^* mice, and continuous PET data were acquired over the following 62 min; a CT scan was performed at the end of the dynamic PET scan. *A*, volumetric renderings of co‐registered images at selected times after bolus administration, for one representative wild‐type mouse (top) and one representative *Sglt1^–/–^* mouse (bottom). PET images are displayed as in Fig. 4 *A*; stomach (st) and intestine (in) are indicated where visible. *B* and *C*, Me‐4FDG absorption (*B*) and excretion into urinary bladder (*C*) as a function of time. Data are for the mice shown in *A*; gastric emptying within the first 2 min was 52% and 60%, and at the end of the experiments was 60% and 80% in the wild‐type mouse and the *Sglt1^–/–^* mouse, respectively. Similar results were obtained in two additional experiments each. These two experiments are shown in Videos S1 and S2.

**Figure 8:**
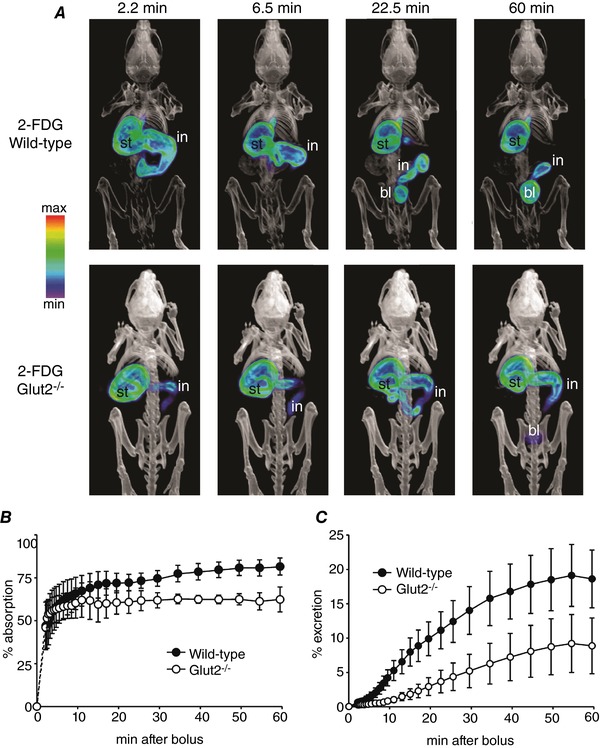
Time course of 2‐FDG absorption and distribution in mice 2‐FDG (300 μCi) was administered by oral gavage into the stomach of anaesthetized wild‐type or *Glut2^–/–^* mice, and continuous PET data were acquired over the following 60 min; a CT scan was performed at the end of the dynamic PET scan. *A*, volumetric renderings of co‐registered images at selected times after bolus administration, for one representative wild‐type mouse (top) and one representative *Glut2^–/–^* mouse (bottom). PET images are displayed as in Fig. 4 *A*; stomach (st), intestine (in) and urinary bladder (bl) are indicated where visible. *B* and *C*, 2‐FDG absorption (*B*) and excretion into urinary bladder (*C*) as a function of time. Data are the mean ± SEM for five wild‐type and three *Glut2^–/–^* mice. Gastric emptying at the end of the experiment was 40 ± 10% in wild‐type and 22 ± 10% in *Glut2^–/^* ^–^ mice (*P* = 0.28, Student's *t* test). PET images are displayed according to the NIH intensity scale for tracer activity, from red (highest), through green (intermediate) to purple (lowest).

**Figure 9:**
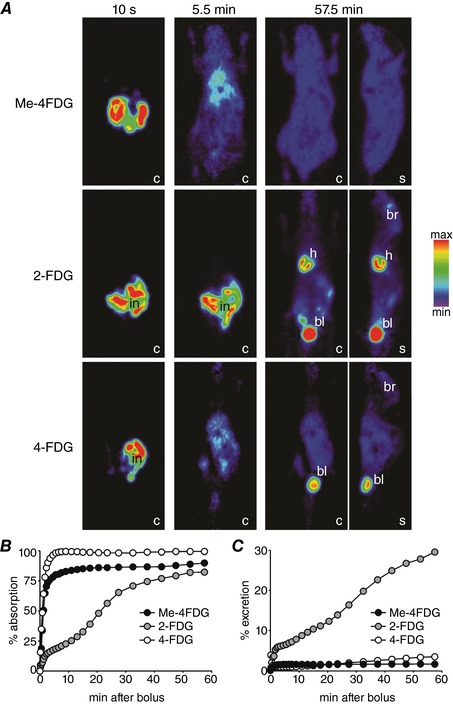
Dynamic Me‐4FDG, 2‐FDG and 4‐FDG microPET scans of control mice following direct sugar delivery into the duodenum Two hundred microcuries of Me‐4FDG (in 5 mm α‐MDG), 2‐FDG (in 5 mm 2‐DG) or 4‐FDG (in 5 mm glucose) was infused into the duodenum of anaesthetized mice, and PET data were continuously acquired for 60 min. *A*, coronal (c) and sagittal (s) PET images at selected times after bolus administration for three representative mice. Images are displayed as in Fig. 4 *A*, and intestine (in), brain (br), heart (h) and urinary bladder (bl) are indicated where visible. *B* and *C*, time course of PET tracer absorption (*B*) and excretion (*C*) for the mice shown in *A*. Results were confirmed in at least one additional experiment for each tracer.

We used dynamic PET scanning to investigate the intestinal absorption of Me‐4FDG, 2‐FDG and 4‐FDG in mice over time, after either orogastric gavage (Figs 7 and 8, and Videos S1 and S2) or direct delivery of the tracers to the duodenum (Fig. 9). The time course of Me‐4FDG absorption in wild‐type and *Sglt1^–/–^* mice after oral gavage is shown in Videos S1 and S2, and Fig. 7. Me‐4FDG can only be clearly observed in the duodenum of the wild‐type mouse during the first 2 min (Video S1), whereas the tracer can be seen throughout the intestine of the Sglt1–/– mouse for the duration of the experiment (Video S2). Quantitative VOI analysis revealed that once in the intestine, absorption of Me‐4FDG in wild‐type mice was quick, and virtually complete within minutes, whereas in *Sglt1^–/–^* mice it was notably delayed, i.e. only ∼50% of the tracer propelled from the stomach was absorbed 1 h after oral delivery (Fig. 7 *B*). Excretion of absorbed Me‐4FDG into the urinary bladder reached a ∼1% plateau by 15 min in wild‐type mice, but it rose linearly with time in *Sglt1^–/–^* mice to ∼2% in 1 h (Fig. 7 *C*). These results, together with the data shown in Figs 3, 4, 5, 6 and Table 1, indicate that SGLT1 plays a key role in the fast absorption of glucose across the small intestine, and that in the absence of SGLT, intestinal glucose absorption is delayed, but not abolished. On the other hand, as anticipated (Sala‐Rabanal *et al*. 2016), the absence of SGLT1 in the kidney results only in a slight loss of Me‐4FDG and 4‐FDG into the urine.

The time course of 2‐FDG absorption in anaesthetized wild‐type and *Glut2^–/–^* mice after oral gavage is shown in Fig. 8. In both groups, 2‐FDG was visible in the small intestine throughout the scan (Fig. 8 *A*), and this was reflected in incomplete tracer absorption, i.e. up to ∼75% in wild‐type and up to ∼60% in *Glut2^−/−^* mice (Fig. 8 *B*). In wild‐type animals, absorbed 2‐FDG was slowly, but significantly, excreted into the urinary bladder (Fig. 8 *C*), consistent with inefficient reabsorption of 2‐FDG from the glomerular filtrate. 2‐FDG excretion was delayed in *Glut2^–/–^* mice (Fig. 8 *C*), presumably due to the lower gastric emptying, i.e. ∼20% in *Glut2^−/−^ vs*. ∼40% in wild‐type mice, and to the lower absorption, i.e. ∼60% *vs*. ∼75%. The PET imaging studies described above revealed that gastric emptying is a major factor in limiting complete glucose absorption in mouse OGTTs, but that once delivered into the intestine, glucose is rapidly absorbed in the duodenum. To confirm this efficient absorption of glucose, we directly infused sugars into the duodenum and followed the time course of absorption by dynamic PET scanning. Figure 9 shows the results for the administration of 200 μl of 0.9% NaCl solutions containing (i) 5 mm α‐MDG and ∼200 μCi Me‐4FDG; (ii) 5 mm 2‐DG and ∼200 μCi 2‐FDG; or (iii) 5 mm glucose and 4‐FDG, i.e. 1 μmol of each sugar. For each experiment, in Fig. 9 *A* we show coronal and sagittal PET images of the mice 10 s, 5.5 min and 57.5 min after delivery, and in Fig. 9 *B* and C the time course of intestinal absorption and renal excretion of each tracer. Complete Me‐4FDG and 4‐FDG absorption was achieved within the first 5 min (Fig. 9 *B*), and there was little (< 3%) excretion of either tracer into the urinary bladder (Fig. 9 *C*). These results are in agreement with our observations in OGTTs, i.e. Me‐4FDG and 4‐FDG are rapidly absorbed after the propulsion of gastric contents into the duodenum, and 2‐FDG is slowly but almost completely absorbed over 1 h.

## Discussion

The purpose of this study was to examine the mechanisms of glucose absorption and distribution during OGTTs using a non‐invasive imaging method, namely positron emission tomography (PET). We are revisiting this topic within the context of the advances in our understanding of the molecular mechanisms of active glucose absorption following the cloning of SGLT1 (Hediger *et al*. 1987; Wright *et al*. 2011) and GLUT2 (James *et al*. 1988; Kayano *et al*. 1988; Thorens *et al*. 1988). While the activity of SGLT1 and GLUT2 readily explains glucose absorption at low concentrations, it is widely recognized that glucose ‘diffusion’ becomes important at high concentrations. It has been proposed that diffusion is due to the rapid recruitment of intracellular GLUT2 into the brush border membrane (Kellett, 2001). However, there is contrary evidence to the GLUT2 hypothesis: for example (i) the passive absorption of glucose, galactose, αMDG and sorbose is similar in rats (Debnam & Levin, 1975), but αMDG and sorbose are not substrates for GLUT2; (ii) glucose OGTTs are similar in a GLUT2‐null patient, wild‐type and *Glut2^–/–^* mice (Stumpel *et al*. 2001; Santer *et al*. 2003; and see Fig. 2); and (iii) GLUT2 plays only a minor role in glucose transport across the brush border membrane even after a high glucose load (Gorboulev *et al*. 2012).

Here we re‐examine the importance of SGLT1 and GLUT2 in glucose absorption during OGTTs using microPET and novel glucose‐specific tracers, i.e. Me‐4FDG, 2‐FDG and 4‐FDG. The advantage of PET is that glucose distribution throughout the entire mouse is followed non‐invasively with high spatial and temporal resolution (Sala‐Rabanal *et al*. 2016). Me‐4FDG is a selective substrate for SGLT1 and 2‐FDG is a selective substrate for GLUTs: the apparent affinity constants of Me‐4FDG for SGLT1 and GLUT2 are 0.06 and 104 mm, respectively, and those of 2‐FDG for GLUT2 and SGLT1 are 7 and > 300 mm, respectively (Sala‐Rabanal *et al*. 2016). Neither Me‐4FDG nor 4‐FDG is metabolized *in vivo*, but 2‐FDG is converted intracellularly to 2‐FDG‐6‐phosphate, which is no longer a substrate for GLUTs. Interestingly, 2‐FDG is not significantly converted to 2‐FDG‐6‐phosphate during intestinal absorption, as 2‐FDG accounts for > 99% of the radioactivity detected in the blood of rats orally dosed with 2‐FDG, and the tracer is accumulated in organs expressing GLUTs, such as brain, heart and kidneys (Yamashita *et al*. 2011; and see also Figs 1 and 9). A limitation of the studies in this work is the challenge to carry out dynamic microPET experiments on fully conscious rodents because of ethical concerns, but here we circumnavigated this problem by conducting brief scans under isoflurane anaesthesia at fixed times after oral gavage. We find that the total amount of sugar absorbed in conscious and anaesthetized mice is the same in these experiments.

We chose to measure absorption under a standard protocol for evaluating glucose homeostasis in mice and man, namely OGTTs. In clinical studies a typical standard test meal consisting of 1–2 g of glucose per kg body weight, i.e. 50–100 g glucose in 240–300 ml of water (1–2.5 m glucose), is consumed after an overnight fast and blood glucose levels are monitored for up to 2 h. In mice, we have performed comparable OGTTs: 2 g kg^−1^ body weight, i.e. 200 μl of 1.3 m glucose (equivalent to ∼10 pmol of sugar per g body weight), was administered directly into the stomach of fasting mice under light isoflurane anaesthesia, and in the presence of ∼300 μCi of tracer where appropriate. In some experiments the mouse was allowed to recover (< 1 min) and roam freely in the cage for up to 1 h before conducting a 10 min microPET scan, whereas in other experiments we conducted 1 h dynamic microPET scans.

The first major finding was that the time course of blood Me‐4FDG and 4‐FDG concentration in conscious mice closely followed that of blood glucose, both in the presence and in the absence of phlorizin in OGTTs (Fig. 3). However, plasma 2‐FDG concentrations did not, and the time course in the presence and absence of phlorizin was close to those for Me‐4FDG and 4‐FDG in the presence of phlorizin. Since Me‐4FDG is a high affinity substrate for SGLTs, but not GLUTs, and since 2‐FDG is a specific substrate for GLUTs but not SGLTs, these results signal the importance of SGLT1 in glucose absorption, and suggest that GLUT2 plays a minor role in OGTTs. This is supported by our OGTT studies in *Glut2^–/–^* mice (Fig. 2) where changes in blood glucose levels were indistinguishable from OGTTs in control mice. In another study of the same *Glut2^−/−^* mouse model there was a significant decrease in the blood glucose level 15 min after gavage with 4 g kg^−1^ body weight (Roder *et al*. 2014). We also note that in another *Glut2^−/−^* mouse model, i.e. tamoxifen‐induced specific Slc2a2 deletion in the small intestine, there was a modest reduction in blood glucose levels in OGTTs (Schmitt *et al*. 2017). OGTTs in *Sglt1^−/−^* mice have shown that blood glucose levels remained relatively flat after gavage (Powell *et al*. 2012; Gorboulev *et al*. 2012)

It is commonly assumed that the change in the OGTT blood glucose profile after phlorizin pre‐treatment reflects the inhibition of glucose absorption by SGLT1. At low concentrations, phlorizin is a specific, competitive inhibitor of SGLT1 (*K*_i_ ∼140 nm; Hummel *et al*. 2012), but non‐specific effects are reported on GLUTs at high concentrations, > 100 μm. The initial glucose concentration in the duodenum in OGTTs is > 1 m, but the concentration of phlorizin is unknown. As discussed below, phlorizin is absorbed and this results in glucose excretion into the urinary bladder, and hence reduction of blood glucose levels, as with oral SGLT2 inhibitors (Gallo *et al*. 2015).

A second finding is that gastric emptying is a rate‐limiting step in glucose absorption in OGTTs in both conscious and isoflurane‐anaesthetized mice. On average, only 60% of the initial stomach contents were propelled into the small intestine of wild‐type, *Sglt1^−/−^* and *Glut2^−/−^* mice in 1 h (Fig. 4 *B*). However, gastric emptying was highly variable, ranging from 10 to 100% (Fig. 4 *B*). These results were comparable to previous reports using MRI imaging (Mudie *et al*. 2014). The efficiency of gastric emptying determines the delivery of glucose into the duodenum, the time course of sugar absorption, and ultimately the time course and peak of the post‐prandial blood glucose concentrations (Holst *et al*. 2016). Thus, it is highly desirable to measure the rate of gastric emptying to interpret OGTTs.

MicroPET imaging of Me‐4FDG, 2‐FDG and 4‐FDG in conscious mice showed that absorption of the sugar that entered the intestine was complete within 1 h, even in mice pre‐treated with phlorizin (Figs 5 and 6). However, at shorter times, i.e. 20 and 35 min, phlorizin partially inhibited absorption (Fig. 6). The decreased inhibition efficiency at later times could be due to hydrolysis of phlorizin to inactive phloretin by the intestinal brush border phlorizin isolactase, but this is unlikely in adult mice as the activity of the enzyme is very low after weaning. Oral phlorizin pre‐treatment resulted in the excretion of sugar into the urinary bladder (Figs 5 and 6). This suggests that phlorizin is absorbed intact from the intestine, filtered at the glomerulus and inhibits SGLTs expressed in the brush border membrane of the proximal tubule (see Ghezzi *et al*. 2017). This implies that the reduction in plasma glucose profiles after phlorizin pre‐treatment (Fig. 3), is, at least in part, due to excretion of glucose into the urine. This is the basis for the SGLT2 inhibitors used to treat diabetes (Gallo *et al*. 2015; Ghezzi *et al*. 2017).

Dynamic microPET studies under isoflurane anaesthesia in OGTT wild‐type mice co‐dosed with Me‐4FDG (e.g. Fig 7 and Video S1) show that the sugar propelled from the stomach into the small intestine was rapidly absorbed in the duodenum, and little Me‐4FDG was detected in the small intestine after the first few minutes. In *Sglt1^−/−^* mice, Me‐4FDG absorption was notably delayed, i.e. ∼50% at 1 h compared to > 90% in wild‐type mice (Fig. 7), and this translated into Me‐4FDG being clearly visible along the intestine throughout the 1 h study (Video S2). In similar experiments with 2‐FDG in control and *Glut2^–/–^* mice (Fig. 8), 2‐FDG absorption was slow in both groups, but 60–75% was absorbed in 1 h (Fig. 8 *B*) and a significant fraction of the absorbed sugar (∼5–15%) was excreted into the urine (Fig. 8 *C*). The 2‐FDG filtered by the kidney glomerulus is not reabsorbed, as 2‐FDG is not a substrate for either SGLT2 or SGLT1 in the proximal tubule. The importance of SGLT1 in glucose absorption was confirmed by the rapid and complete absorption of 5 mm Me‐4FDG and 4‐FDG delivered directly into the duodenum (Fig. 9). Absorption of 2‐FDG directly introduced into the duodenum was slow, but practically complete after 1 h.

In OGTTs we conclude that glucose is rapidly absorbed by SGLT1 in the early duodenum since (i) no Me‐4FDG is observed in the duodenum within minutes of gavage, (ii) Me‐4FDG is observed in the intestine at early times after phlorizin treatment, and (iii) Me‐4FDG is observed all along the small intestine in *Sglt1^−/−^* mice. In contrast, 2‐FDG is observed all along the small intestine. In the mouse SGLT1 is expressed in the brush boarder membrane from duodenum to ileum (Madunic *et al*. 2017) and this suggests that glucose released from complex carbohydrates may be absorbed all along the small intestine.

In summary, these studies show that SGLT1 plays an important role in the fast absorption of glucose during OGTTs in mice, whereas GLUT2 is of minor importance. In the absence of SGLT1, glucose is slowly absorbed in the intestine, i.e. up to 50% in 1 h. This may account for the small, slow increase in blood glucose in the OGTTs carried out in mice pre‐treated with phlorizin (Fig. 3). What is the mechanism of glucose absorption in the absence of SGLT1 or GLUT2? There are at least three suggestions. (i) Passive diffusion through enterocytes. This is reasonable as we estimate the rate of passive absorption at 100 mm glucose to be ∼500 μmol h^−1^, given the vast surface area of the small intestine, i.e. ∼1.5 m^2^ (Casteleyn *et al*. 2010), and a glucose permeability of ∼1 × 10^−7 ^cm s^−1^ (Bindslev & Wright, 1976). (ii) Diffusion through the paracellular pathway. This is less compelling than diffusion through the cellular pathway given the structure, permeability properties and small relative area of the tight junctions. Although there is strong evidence for ion permeation through the paracellular route, there is only circumstantial evidence for water and small‐non‐electrolyte permeation. And (iii) transport through low affinity, non‐selective carriers for monosaccharides expressed in enterocytes; however, no such transporters have been identified so far. A similar issue occurs with glucose release from the liver of mice where GLUT2 plays a minor role in glucose exit (Guillam *et al*. 1998), and we and others did not observe any difference between steady state 2‐FDG levels (% ID g^−1^) in control and *Glut2^−/−^* mice irrespective of the route of delivery, i.v. or gavage (Sala‐Rabanal *et al*. 2016; Schmitt *et al*. 2017). For both liver and intestine Thorens has proposed that glucose exits by an exocytosis mechanism involving glucose and de‐phosphorylation (Guillam *et al*. 1998; Stumpel *et al*. 2001), but this cannot explain Me‐4FDG or αMDG absorption as neither are phosphorylated.

How do we interpret OGTTs in terms of sugar absorption? This is a non‐trivial task owing to the rate‐limiting effect of gastric emptying and plasma insulin‐dependent disposal of glucose into organs such as the liver, brain and heart, and gluconeogenesis (Rizza *et al*. 2016). However, using the Me‐4FDG microPET data, we can estimate the rate of glucose absorption. Given that 60 mg (330 μmol) is gavaged into the stomach and 60% of this is propelled into the duodenum in 1 h (36 mg or 200 μmol), 36 mg (200 μmol) is absorbed in 1 h. SGLT1 accounts for the rapid absorption in the duodenum, and this undoubtedly contributes to the relatively fast rise in plasma glucose levels. In the presence of phlorizin (Fig. 3), or the absence of SGLT1 (Powell *et al*. 2012; Gorboulev *et al*. 2012), there is no significant increase in blood glucose or Me‐4FDG levels, but there is a slow absorption of glucose amounting to at least 36 mg (100 μmol) over 1 h. This slow absorption is not sufficient to cause a significant rise in blood glucose level due to disposition of glucose in organs throughout the body. In the case of phlorizin, the absence of a rise in blood glucose is in part due to the inhibition of reabsorption from the glomerular filtrate (Fig. 6). This calls for caution in the interpretation of the effect of other SGLT1 inhibitors on OGTTs.

Clearly, our studies do not address other factors such as diet and fasting, or incretin secretion on OGTTs. It is well established that during OGTTs glucose stimulates the release of incretins though SGLT1 glucose sensing (Gorboulev *et al*. 2012; Roder *et al*. 2014; Kuhre *et al*. 2017). However, we are not aware that these signalling molecules influence gastric emptying or glucose absorption during the 60 min OGTTs, but we do know that insulin does not affect SGLT1 activity in HEK293 cells (Ghezzi & Wright, 2012).

What is the importance of GLUT2 expression on the basolateral membranes of the intestinal epithelium, if it does not play a major part in dietary glucose absorption? One possibility is the supply of glucose to the enterocytes from the blood. Enterocytes have a high requirement for ATP to drive salt and water absorption, and in the absence of dietary carbohydrate, glucose from the blood may meet the demand for ATP synthesis.

Finally, there is a marked difference between the importance of GLUT2 in intestinal and renal glucose transport in that GLUT2 plays a minor role in intestinal glucose absorption, whereas GLUT2 in the kidney proximal tubule basolateral membrane is essential for glucose reabsorption from the glomerular filtrate (Sala‐Rabanal *et al*. 2016). This may be due to large differences in fluid transit times along the proximal tubule and small intestine.

## Additional information

### Author's present address

V. Kepe: Department of Nuclear Medicine, Cleveland Clinic, Cleveland, OH 44195, USA.

### Competing interests

All authors declare that they have no conflicts of interest.

### Author contributions

M.S‐R., C.G., E.M.W. and J.R.B. were responsible for the study concept and design. M.S‐R., B.A.H., C.G. and J.L. were responsible for the acquisition of data. M.S‐R., C.G. and E.M.W. were responsible for data analysis and interpretation; J.L. and V.K. were responsible for chemical design, synthesis and analysis. M.S‐R., C.G. and E.M.W. were responsible for manuscript preparation and revision. All authors have read and approved the final version of this manuscript and agree to be accountable for all aspects of the work in ensuring that questions related to the accuracy or integrity of any part of the work are appropriately investigated and resolved. All persons designated as authors qualify for authorship, and all those who qualify for authorship are listed.

### Funding

This work was supported by grants from the National Institutes of Health (Grants RO1‐DK19567 (E.M.W.) and RO1‐DK077133 (E.M.W.) and the Elizabeth and Thomas Plott Endowed Chair in Gerontology (J.R.B.). Additional funding was provided by a Pilot and Feasibility Grant from the UCLA Digestive Diseases Research Centre, and the Mitzi and William Blahd Pilot Research Grant from the Society of Nuclear Medicine (Sala‐Rabanal).

**Translational perspective**

These mouse microPET studies of glucose absorption in mice clearly provide a foundation for similar PET studies on sugar absorption in healthy human subjects and patients. Such studies may provide insights into the importance of gastric emptying, the role of SGLT1 and GLUT2 in glucose absorption in health and disease, and the influence of drugs, e.g. SGLT1 inhibitors being developed to treat type 2 diabetes mellitus. 2‐FDG PET is already firmly entrenched in the clinic for the detection and staging of cancer, as well as cardiac and neurological diseases, and this means that the technology is already in place.

## Supporting information

Disclaimer: Supporting information has been peer‐reviewed but not copyedited.

**Video S1**: Distribution of the tracer was followed for 1 h. Frames were collected every 2 min and the movie was created using Amide. The maximum and minimum thresholds of %ID g^−1^ used are 12 and 0, respectively.

**Video S2**: Distribution of the tracer was followed for 1 h. Frames were collected every 2 min and the movie was created using Amide. The maximum and minimum thresholds of %ID g^−1^ used are 12 and 0, respectively.
